# Chemical Composition and Nutritional Profile of Quinoa Sourdough Enriched with Quinoa Malts

**DOI:** 10.3390/molecules30173653

**Published:** 2025-09-08

**Authors:** Agata Wojciechowicz-Budzisz, Alan Gasiński, Witold Pietrzak, Ewa Pejcz, Marzena Styczyńska, Joanna Harasym

**Affiliations:** 1 Department of Biotechnology and Food Analysis, Wroclaw University of Economics and Business, 53-345 Wrocław, Poland; joanna.harasym@ue.wroc.pl; 2 Department of Fermentation and Cereals Technology, Wroclaw University of Environmental and Life Sciences, 51-630 Wrocław, Poland; alan.gasinski@upwr.edu.pl (A.G.); witold.pietrzak@upwr.edu.pl (W.P.); 3 Department of Human Nutrition, Wroclaw University of Environmental and Life Sciences, 51-630 Wrocław, Poland; marzena.styczynska@upwr.edu.pl; 4 Adaptive Food Systems Accelerator–Science Centre, Wroclaw University of Economics and Business, 53-345 Wroclaw, Poland

**Keywords:** quinoa, sourdough, malt, quality, health-enhancing properties

## Abstract

This study investigated the combined effects of quinoa malt addition (0%, 5%, 10%) and grain variety (white, red, black) on the nutritional and sensory properties of quinoa sourdoughs. Quinoa malt supplementation significantly (*p* < 0.05) enhanced fermentation characteristics, increasing titratable acidity from 20.0–20.4 to 21.2–23.8 mL NaOH/10 g and dynamic viscosity up to 733 ± 5.59 mPa·s compared to 474–611 mPa·s in controls. Malt enrichment expanded the volatile profile from predominantly alcohols and acids to include 25 distinct compounds spanning esters, terpenes, aldehydes, phenols, and furans, creating more complex aromatic profiles. Lactic acid production increased significantly in all malted samples, reaching 12.92 ± 0.00 g/kg in black quinoa with 10% malt. Black quinoa sourdoughs exhibited superior nutritional density with the highest protein (17.3 ± 0.1%), total dietary fiber (17.94 ± 0.14%), potassium (7896 ± 176 mg/kg), and manganese (55.65 ± 0.47 mg/kg) contents (*p* < 0.05). White quinoa variants demonstrated the highest acidity (pH 4.28 ± 0.01) and mineral bioavailability (magnesium: 5371 ± 70 mg/kg), while red quinoa achieved maximum viscosity (733 ± 5.59 mPa·s) and zinc content (38.08 ± 0.26 mg/kg). Volatile compound distribution varied significantly by variety, with white quinoa favoring ester and terpene formation, red quinoa promoting aldehydes and terpenes, and black quinoa accumulating phenols and furans. These findings demonstrate that strategic combination of quinoa variety selection and malt optimization can produce functionally enhanced, gluten-free sourdoughs with targeted nutritional and sensory characteristics for specialty bakery applications.

## 1. Introduction

Cereals and pseudocereals serve as essential staple foods and are also key raw materials in the production of various products for human consumption. The quality and sensory characteristics of these finished products are significantly affected by the physicochemical properties and volatile composition (e.g., aroma compounds) of the raw ingredients [1].

Quinoa (*Chenopodium quinoa* Willd.), a member of the *Chenopodiaceae* family alongside spinach and beet, is native to South America and was domesticated by the Andean peoples [2]. Known for its remarkable adaptability to diverse weather, climate, and soil conditions, quinoa has traditionally been regarded as a sacred plant, valued for its rich protein content and exceptional balance of essential amino acids [3]. Indigenous populations in South America have long relied on quinoa as a staple food, recognizing its nutritional benefits [2]. Quinoa’s seeds are particularly notable as the most economically and scientifically significant part of the plant. They feature a balanced protein profile rich in sulfur-containing amino acids, along with lysine and lipids, making quinoa an excellent dietary source. More recently, quinoa has gained global recognition as a functional food, praised for its high levels of proteins, fibers, lipids, vitamins, minerals, and amino acids. Additionally, it is abundant in phytochemicals such as saponins, phytosterols, phytoecdysteroids, phenolic compounds, and bioactive peptides, further enhancing its health-promoting properties [4,5]. Research highlights the exceptional nutritional value of quinoa, attributed to its balanced composition and high content of proteins, minerals, fiber, antioxidants, and vitamins. Quinoa seeds typically contain 13.8–16.5% protein, with an average of 15%. Of this, albumins constitute approximately 35%, and globulins account for 37%. Notably, quinoa provides all nine essential amino acids—phenylalanine, methionine, histidine, isoleucine, valine, leucine, lysine, threonine, and tryptophan—crucial for human growth and development [6].

Quinoa is naturally gluten-free due to the absence of gliadins and related protein fractions, making it an excellent option for individuals with celiac disease. Additionally, quinoa contains lysine, methionine, vitamins B6 and B9, iron, and calcium. Its dietary fiber content ranges from 7.0 to 9.7%, with some reports suggesting values as high as 3.0–52.0% depending on the variety. Quinoa is also rich in phenolic compounds, particularly flavonoids, which provide significant health benefits. Research confirms that quinoa is non-toxic and has positive effects on human health, particularly in supporting gastrointestinal function, metabolism, and cardiovascular health [6,7,8,9].

Owing to its remarkable nutritional and biological properties, quinoa is often referred to as the “golden grain”. Moreover, NASA has recognized it as an ideal crop for astronaut nutrition, underscoring its versatility and importance [10].

Sourdough fermentation offers a versatile approach to enhancing the technological, nutritional, functional, and sensory qualities of both wheat and non-wheat flours. Specifically, it improves dough workability, bread structure, and the organoleptic and nutritional characteristics of the raw flours. Additionally, it boosts the content of biogenic compounds, enhances mineral bioavailability, reduces anti-nutritional factors, and lowers the glycemic response [11].

Fermentation processes rely on specific determinants that must be carefully controlled to produce standardized and desirable products [12,13]. Among these, the type of flour plays a crucial role, influencing the technological properties, nutritional value of baked goods, and microbial fermentation. This impact is driven by the level and type of fermentable carbohydrates, nitrogen sources, and growth factors present in the flour [14]. To enhance sourdough quality, innovative approaches are being explored, including the incorporation of quinoa malts.

Malt is a processed grain that undergoes germination (sprouting) followed by drying in a procedure known as malting. This process activates enzymes, particularly amylase, which converts the grain’s starches into simpler sugars, making them more readily available for yeast during fermentation. It also reduces antinutrients like phytate and saponins, improving mineral bioavailability and sensory properties. Incorporating quinoa malt into sourdough enhances its flavor profile, nutritional value, and fermentation dynamics, giving bakers a unique opportunity to craft breads with distinctive taste and texture. Malt enriches the flavor of sourdough with mild sweetness and toasty or nutty undertones while contributing to a softer crumb and improved texture. Additionally, quinoa malt provides a healthier, high-protein alternative, elevating the overall nutritional profile of the bread. The additional sugars from malt fuel yeast activity, promoting better rise and structure, while also encouraging the growth of beneficial lactic acid bacteria, which enhances the sourness and complexity of sourdough flavors. Furthermore, malt deepens the crust color, creating a beautifully caramelized, golden-brown loaf with a richer, more nuanced taste. By incorporating malt, bakers can achieve superior-quality sourdough with exceptional flavor and visual appeal [1,15,16,17,18,19,20,21,22,23,24,25].

Although both sourdough and quinoa have been widely studied, limited attention has been given to the enrichment of sourdough with malts derived from this pseudocereal. Existing studies on quinoa-based sourdoughs focus primarily on whole or flaked grains without sprouting, leaving a gap in understanding how quinoa malt—produced through controlled germination—affects fermentation performance, aroma development, and nutritional enrichment compared to its non-sprouted counterpart. Quinoa, renowned for its exceptional nutritional profile, has attracted global attention as a functional food ingredient, particularly in gluten-free formulations. Incorporating quinoa malt into sourdough fermentation represents a promising strategy to improve both the nutritional value and sensory attributes of gluten-free bakery products.

This study addresses the identified research gap by evaluating the influence of quinoa malt flours, derived from three varieties—white, red, and black—on the quality, fermentation behavior, and health-related properties of wholemeal quinoa sourdoughs. Specifically, we examine how malt addition affects acidity, dietary fiber fractions, protein content, mineral composition, and volatile compound profiles.

We hypothesize that the ratio of quinoa malt addition and varietal differences will jointly modulate the metabolite profile and nutritional quality of quinoa-based sourdoughs, with sprouted quinoa enhancing fermentation efficiency, reducing antinutrients, and improving the sensory characteristics compared to non-malted flours.

## 2. Results and Discussion

Table 1 presents the physical and chemical properties of six samples (WQ—white quinoa, RQ—red quinoa, BQ—black quinoa, WQM—white quinoa malt, RQM—red quinoa malt, BQM—black quinoa malt), including falling number, protein content, moisture, ash, fat, dietary fiber fractions (SDF—soluble dietary fiber, IDF—insoluble dietary fiber, TDF—total dietary fiber), and colorimetric parameters (L*, a*, and b*) and reveals substantial variability in their technological and nutritional attributes. These differences were primarily influenced by both the varietal characteristics of quinoa and the modifications introduced by malting. The falling number, indicating starch quality and enzyme activity, showed pronounced differences among samples and ranged from 116 ± 6 s (RQM) to 2091 ± 105 s (BQ). WQ had a relatively high falling number (1712 ± 86 s), while WQM (584 ± 29 s) and BQM (322 ± 16 s) showed moderate values. RQ and RQM exhibited significantly lower values, suggesting greater enzymatic activity or reduced starch stability compared to BQ and WQ. Unmodified black quinoa (BQ) displayed an exceptionally high value, indicating minimal enzymatic degradation of starch and high starch stability. Since α-amylase is critical for breaking down starch into fermentable sugars, this enzymatic inactivity suggests that native BQ flour provides limited available substrates for yeast and lactic acid bacteria during fermentation. As a consequence, the reduced amylolytic potential may hinder microbial metabolism and acid production in sourdough systems lacking external enzyme sources. Malting generally reduced the falling number in all variants, consistent with reports that germination enhances endogenous enzyme activity, which degrades starch and reduces pasting resistance [15,26]. Protein content varied significantly across samples, with the highest in BQ (16.3 ± 0.1%) and BQM (16.4 ± 0.2%), supporting previous findings that darker-colored quinoa varieties tend to be richer in protein [27,28], and the lowest in WQ (13.9 ± 0.1%). Modifications increased protein content slightly in WQM and RQM compared to their unmodified forms, reflecting potential processing or enrichment effects. This may be attributed to enzymatic mobilization and concentration effects during germination and drying [29]. Moisture content showed a wide range, with the highest observed in WQ (11.0 ± 0.0%), likely due to lower drying efficiency or higher hygroscopicity of the white variety. In contrast, malted samples—especially BQM (3.1 ± 0.0%)—had much lower moisture levels, reflecting the desiccation step typical of malt preparation [30]. Ash content, indicative of mineral levels, ranged from 2.17 ± 0.03% (RQM) to 2.70 ± 0.01% (BQ). Modifications led to slight reductions in ash content in WQM and RQM compared to their base samples, possibly due to leaching or redistribution during soaking and germination. Fat content was the highest in BQM (7.45 ± 0.07%) and lowest in WQ (5.80 ± 0.14%). Modifications appeared to have minimal effect on fat content across samples. The increase observed in BQM could be linked to concentration effects or lipid mobilization during sprouting. As quinoa is known for its relatively high lipid content compared to other pseudo-cereals, the fat levels observed remain nutritionally relevant [26,27].

In terms of dietary fiber, BQ and BQM had the highest total dietary fiber content (16.02 ± 0.63% and 14.86 ± 0.84%, respectively), driven largely by high levels of insoluble fiber (IDF), and the lowest in WQ and WQM (9.20 ± 0.21% and 9.01 ± 0.18%, respectively). This aligns with previous studies showing darker quinoa varieties to be richer in structural polysaccharides and fiber-associated phenolic compounds [28,31]. Soluble dietary fiber (SDF) was more evenly distributed across samples but tended to be higher in RQ (3.34 ± 0.03%) and BQM (2.90 ± 0.02%). The elevated fiber levels in malted samples support the notion that germination can increase fermentable fiber fractions by degrading cell wall components and modifying soluble polysaccharides [1].

Colorimetric analysis revealed that quinoa variety and malting significantly influenced visual attributes. Lightness (L*) was highest in WQ (89.49 ± 0.22) and WQM (88.68 ± 0.01) and lowest in RQ (68.53 ± 0.30) and RQM (73.56 ± 0.04), consistent with pigment intensity in red quinoa. Interestingly, malting slightly reduced L* values across all variants, likely due to Maillard browning or pigment changes during kilning [1]. The a* parameter (red-green axis) was most negative in WQM (–5.22 ± 0.01), indicating a greener hue, while RQ had a slightly positive a* (0.24 ± 0.09), indicating reddish tones typical of anthocyanin-rich grains. Yellow-blue balance (b*) was highest in WQM (19.67 ± 0.28), suggesting that malting intensified yellow coloration—likely due to pigment concentration or heat-induced chromophore formation.

Overall, these findings demonstrate that both quinoa variety and malting modification significantly impact the physical and chemical properties of quinoa-based ingredients, as confirmed by ANOVA and Duncan’s post hoc test. The observed changes in starch functionality (falling number), protein and fiber content, and color parameters suggest potential for targeted functional applications in fermented bakery products. In particular, black quinoa and its malted form emerge as nutritionally dense, high-fiber raw materials with favorable protein content and unique color attributes, while white quinoa malt provides a visually appealing base with enhanced biofunctional potential.

Table 2 presents the mineral content of six samples (WQ, RQ, BQ, WQM, RQM, BQM), specifically sodium (Na), calcium (Ca), potassium (K), magnesium (Mg), copper (Cu), manganese (Mn), iron (Fe), and zinc (Zn). The mineral composition of the quinoa samples and their malted counterparts revealed marked differences depending on both quinoa variety and processing. These differences reflect not only the inherent genetic variation among quinoa cultivars but also the impact of malting on mineral solubility, retention, and redistribution. Sodium (Na) content showed the most pronounced increase following malting, particularly in WQM, which exhibited the highest concentration (59.97 ± 0.99 mg/kg), almost threefold greater than its unmodified counterpart (WQ, 22.08 ± 0.25 mg/kg). This suggests that sodium may have been introduced during soaking or retained more efficiently during germination and drying. In contrast, both RQ and BQ exhibited low sodium levels (8.25 ± 0.37 and 6.88 ± 0.21 mg/kg, respectively), indicating varietal differences or lower absorption capacity for sodium during processing. Similar trends of sodium accumulation during germination have been previously observed in pseudocereals and legumes subjected to water-based treatments [32]. Calcium concentrations ranged from 45.90 ± 3.98 mg/kg (WQ) to 57.60 ± 2.99 mg/kg (WQM), with slight increases observed in malted samples.

Although the differences between modified and unmodified samples were not always statistically significant, the overall trend suggests that malting may slightly enhance calcium bioavailability through phytate degradation or improved solubilization [23]. Potassium (K) was the most abundant macroelement across all samples, with BQ (7477 ± 64 mg/kg) and BQM (6577 ± 76 mg/kg) showing particularly high levels. These values are consistent with previous studies highlighting black quinoa as a rich source of potassium [28]. WQ and WQM had substantially lower potassium content (5090 ± 94 mg/kg and 5024 ± 40 mg/kg, respectively) and the malting process appeared to have negligible effect on this element in the white quinoa group. Potassium is known to be relatively stable during hydrothermal processing and germination. The high potassium content in BQ and BQM indicates that this sample group may be particularly rich in this essential mineral.

Magnesium (Mg) content was significantly elevated in WQ (4897 ± 80 mg/kg) and WQM (4961 ± 19 mg/kg), with increases also noted in BQM compared to their native forms. This may be linked to mineral mobilization during germination or breakdown of anti-nutritional factors, such as phytates, that bind magnesium in raw seeds [33].

Given the nutritional importance of magnesium in metabolic and neuromuscular function, quinoa malt—particularly WQM—may serve as a valuable dietary source. Copper concentrations ranged from 4.50 ± 0.07 mg/kg (RQ) to 6.04 ± 0.38 mg/kg (WQM), indicating a varietal and process-dependent accumulation. WQM exhibited significantly higher copper levels compared to all other samples, while RQ had the lowest value. Modification slightly increased copper content in WQM, but changes were minimal in other samples. Manganese (Mn) was strongly associated with darker quinoa varieties, with the highest concentrations in BQ and BQM (52.64 ± 0.42 and 53.87 ± 0.88 mg/kg, respectively). In contrast, WQ and WQM showed values less than half of those observed in the black quinoa group (24.26 ± 0.60 and 23.63 ± 0.23 mg/kg, respectively). These results corroborate earlier findings that pigment-rich quinoa varieties tend to be more mineral-dense, potentially due to co-localization of pigments and micronutrients in the seed coat [31]. Modifications slightly increased Mn in RQM and BQM but had little effect on WQM. Iron (Fe) content showed little variability across samples, with all values falling between 83.41 ± 1.78 mg/kg (BQ) and 103.62 ± 8.35 mg/kg (RQ). There were no statistically significant differences between samples. This may reflect the fact that iron is tightly bound within the seed matrix and less affected by the mild processing steps involved in malting [30]. Nevertheless, all quinoa samples displayed high iron content compared to many cereals, affirming quinoa’s value in addressing micronutrient deficiencies. Zinc (Zn) concentrations showed moderate variability and ranged from 29.22 ± 0.20 mg/kg (BQ) to 36.96 ± 0.69 mg/kg (RQM). Malting generally enhanced zinc content in WQM and RQM, potentially through phytate degradation, which can release bound zinc. However, BQM showed slightly lower zinc levels than its native form (BQ), indicating possible varietal differences in zinc retention during processing.

In summary, both quinoa variety and malting significantly influenced mineral composition, as confirmed by ANOVA and Duncan’s post hoc test. Malting most strongly affected sodium, magnesium, and zinc content, while potassium and iron remained relatively stable. Black quinoa and its malted form were richest in potassium, manganese, and iron, while white quinoa malt (WQM) demonstrated the highest levels of sodium, calcium, magnesium, and copper. These findings suggest that quinoa malt—particularly WQM and BQM—could serve as functional ingredients to enhance the mineral density of fermented cereal products, especially for populations at risk of micronutrient deficiencies.

The physicochemical characterization of the quinoa sourdough samples—prepared from white (WQS), red (RQS), and black (BQS) quinoa, each supplemented with 0%, 5%, or 10% quinoa malt—revealed significant differences (*p* < 0.05) in acidity, color, viscosity, protein, ash, and dietary fiber profiles (Table 3). These variations reflect the combined effects of quinoa variety, malt addition, and fermentation dynamics. Acidity parameters (pH and TTA-total titratable acidity) varied significantly among the samples. The pH ranged from 4.28 ± 0.01 (WQS10%M—white quinoa sourdough with 10% of white quinoa malt) to 4.47 ± 0.00 (BQS), indicating mild acidification across all sourdoughs. A slight pH decrease was observed in all malted samples, especially in WQS5%M—white quinoa sourdough with 5% of white quinoa malt and WQS10%M, suggesting enhanced microbial activity and organic acid production during fermentation. Notably, WQS10%M exhibited the lowest pH and highest total titratable acidity (TTA = 23.8 ± 0.2), which may indicate higher fermentative potential or substrate availability in white quinoa with malt supplementation. Conversely, the lowest TTA values were found in RQS and BQS variants (around 20.0), suggesting that red and black quinoa may have higher buffering capacity or lower fermentability, as previously observed in colored quinoa due to their phenolic and saponin content [28,31]. Compared to traditional cereal-based malt sourdoughs (e.g., wheat and rye), quinoa-based sourdough systems display distinct fermentation behavior and nutritional advantages. In conventional systems, malted wheat or rye enhances fermentability through robust endogenous amylase activity, rapidly breaking down starches into fermentable sugars [32,33]. However, quinoa—particularly black quinoa—exhibits markedly lower native α-amylase activity (e.g., falling number > 2000 s), requiring external enzymatic input such as malt addition to achieve similar fermentation performance. This study demonstrates that quinoa malt can fulfill that role, especially at 10% addition, leading to increases in TTA.

Colorimetric parameters (L*, a*, b*) were strongly influenced by quinoa variety and malt addition. WQS samples showed the highest lightness (L* = 89.5 ± 0.9), reflecting their naturally pale color, while BQS samples were markedly darker (L* = 64.1 ± 0.4), as expected from their seed coat pigmentation. The a* values ranged from strongly negative in WQS (−6.09 ± 0.09) to positive in RQS5%M—red quinoa sourdough with 5% of red quinoa malt (+1.36 ± 0.08), suggesting a redder hue possibly associated with pigment retention or Maillard browning reactions during fermentation in red quinoa samples. The b* values, representing yellowness, peaked in WQS10%M (21.95 ± 0.18), indicating that malting may intensify yellow pigments (such as flavonoids or carotenoid-like compounds) or cause changes in matrix color due to biochemical transformations.

Dynamic viscosity ranged from 474 ± 5.59 mPa·s (BQS) to 733 ± 5.59 mPa·s (RQS10%M—red quinoa sourdough with 10% of red quinoa malt), with malt-supplemented samples generally showing increased viscosity. This could be linked to partial starch hydrolysis, fiber solubilization, or protein denaturation during germination and fermentation [22,29,30,34]. The highest viscosity in RQS10%M suggests that red quinoa with added malt may form more structured or gelatinous dough matrices, which could positively affect dough handling and bread texture.

Protein content ranged from 14.5 ± 0.2% (WQS5%M) to 17.3 ± 0.1% (BQS). Black quinoa and its derivatives consistently showed higher protein levels, which aligns with previous studies reporting elevated protein content in darker quinoa varieties [26,27]. Interestingly, malt addition did not consistently increase protein content—samples showed a slight decline post-modification, possibly due to proteolysis or dilution by enzymatic activity.

Ash content, representing the total mineral fraction, ranged from 2.45 ± 0.02% (RQS10%M) to 2.84 ± 0.03% (BQS). The unmodified and malted BQS samples retained the most minerals, likely due to seed coat concentration effects or reduced leaching during processing. Ash content appeared relatively unaffected by malting, indicating stability of mineral composition across the fermentation process. The analysis of dietary fiber fractions revealed marked differences. Values of SDF ranged from 2.28 ± 0.44% (WQS) to 4.01 ± 0.07% (RQS), with most malted samples exhibiting modest increases. This is consistent with the partial enzymatic breakdown of insoluble fiber into soluble components during germination, enhancing water solubility and fermentability. Insoluble dietary fiber (IDF) was highest in BQS (15.62 ± 0.18%) and remained relatively high in BQS5%M (black quinoa sourdough with 5% of red quinoa malt) and BQS10%M (black quinoa sourdough with 10% of red quinoa malt), suggesting that black quinoa retains structural polysaccharides despite modification. The lowest IDF value was observed in WQS10%M (4.16 ± 0.14%), indicating that white quinoa is more prone to fiber breakdown during malting and fermentation. As a result, total dietary fiber (TDF) was highest in BQS10%M (18.42 ± 0.10%) and lowest in WQS10%M (6.73 ± 0.17%), showing a strong correlation between quinoa variety, fiber composition, and processing sensitivity. Nutritionally, quinoa malt sourdoughs offer superior protein quality, with all nine essential amino acids, and significantly higher levels of dietary fiber, magnesium, and zinc than commonly observed in wheat or rye sourdough [14]. Additionally, quinoa contains unique bioactive compounds such as saponins, phytoecdysteroids, and flavonoids, which are largely absent in traditional cereals [26,27,35].

In summary, the ANOVA data demonstrate that quinoa variety is a key determinant of sourdough composition, with black quinoa providing superior nutritional properties, especially in terms of protein and fiber content. Malt supplementation and fermentation further modulate these characteristics, often enhancing acidity, viscosity, and soluble fiber content. The combination of black quinoa and malt appears particularly promising for the development of fiber-enriched functional baked products, while white quinoa sourdoughs with malt show improved color and fermentation profiles but lower structural fiber retention.

Table 4 reports mineral concentrations (mg/kg) for nine samples: WQS, WQS5%M, WQS10%M, RQS, RQS5%M, RQS10%M, BQS, BQS5%M, and BQS10%M. Sodium content varied significantly across the samples, with the highest levels found in WQS5%M (80.95 ± 2.16 mg/kg) and the lowest in BQS5%M (53.92 ± 1.10 mg/kg). The WQS group consistently showed higher sodium concentrations than RQS and BQS groups. This pattern may result from varietal differences in sodium retention or absorption during soaking and fermentation. In particular, the decrease in sodium content in the BQS group after modification suggests leaching or breakdown of sodium-binding components during processing [22,34]. Moreover, as no additional salt was added, the observed variations likely reflect the impact of germination and fermentation on mineral availability and mobility. Calcium (Ca) levels ranged from 18.89 ± 1.53 mg/kg (BQS5%M) to 46.72 ± 0.65 mg/kg (WQS5%M). The WQS group maintained higher calcium content across all treatments, suggesting that white quinoa may have a higher initial calcium level, which was relatively unaffected by malting. This may be due to differences in seed coat permeability or mineral-binding components [31]. No substantial losses were observed with malt addition, indicating that calcium was retained effectively during processing. Potassium (K) was the most abundant macroelement across all samples, with black quinoa sourdoughs (BQS) demonstrating the highest concentrations (up to 7896 ± 176 mg/kg), even after malting. In contrast, WQS samples showed significantly lower potassium levels, with WQS10%M recording the lowest value (5583 ± 127 mg/kg). This supports previous findings that darker quinoa varieties, particularly black quinoa, are richer in potassium and other essential minerals [28]. The slight decline observed in malted samples may be due to solubilization and loss of minerals during soaking or enzyme activation phases of malting. Magnesium (Mg) followed a different trend: it was highest in unmodified WQS (5371 ± 70 mg/kg) and slightly decreased with malt supplementation. The RQS group displayed the lowest magnesium values across all treatments. This indicates that white quinoa is a superior source of magnesium, although partial losses may occur during malting due to increased mineral solubility or dilution effects. Copper (Cu) concentrations were highest in WQS5%M (6.16 ± 0.11 mg/kg) and generally lower in BQS samples (lowest in BQS5%M: 4.79 ± 0.09 mg/kg). The elevated copper levels in WQS, particularly after modification, could suggest that malting enhances mineral bioavailability, possibly through phytate degradation. These findings align with studies that report increased trace mineral extractability in germinated pseudocereals [22,34]. Manganese (Mn) content showed a strong varietal effect: BQS and BQS10%M had the highest levels (55.65 ± 0.47 and 57.62 ± 0.71 mg/kg, respectively), while WQS10%M recorded the lowest (25.62 ± 0.86 mg/kg). The high Mn content in black quinoa is consistent with previous observations linking seed pigmentation to mineral density, as manganese often co-occurs with polyphenolic compounds in pigmented seeds. Mn is a crucial cofactor for various enzymatic processes, including those involving polyphenol oxidase (PPO), which plays a role in the oxidation of phenolic compounds. Given that black quinoa is known to contain higher levels of polyphenols, the increased manganese content may support enhanced PPO activity, potentially contributing to the antioxidant potential and darker coloration of the sourdough matrix [31]. Malting did not negatively affect Mn levels and may have slightly concentrated this element in darker quinoa types. Iron (Fe) concentrations ranged narrowly between 90.76 ± 1.14 and 106.17 ± 1.05 mg/kg, showing no statistically significant differences in ANOVA test among the samples. This suggests that iron content is relatively stable across processing and quinoa variety, likely due to its strong binding within the seed matrix [30]. Nevertheless, the overall iron levels remained high, supporting the role of quinoa sourdoughs as valuable sources of dietary iron. Zinc (Zn) content was highest in RQS and RQS10%M (37.83 ± 0.64 and 38.08 ± 0.26 mg/kg, respectively) and lowest in BQS samples (30.97 ± 0.62–31.36 ± 0.78 mg/kg). The RQS group demonstrated the best zinc retention or availability, especially after malt supplementation, possibly due to germination-induced breakdown of antinutritional compounds such as phytates that limit zinc bioavailability. On the other hand, BQS samples showed consistently lower zinc levels, pointing to varietal limitations or mineral redistribution during processing.

In conclusion, the ANOVA data clearly indicate that quinoa variety plays a dominant role in determining mineral content, while malt supplementation introduces more nuanced, nutrient-specific changes. Black quinoa sourdoughs (BQS) are particularly rich in potassium, manganese, and iron, while white quinoa (WQS) offers higher levels of magnesium, calcium, and copper, especially when combined with 5% malt. These distinctions support the formulation of quinoa-based fermented foods targeted at specific mineral enrichment, depending on nutritional goals.

Gas chromatography and mass spectrometry (GC-MS) allowed for identification and quantification of 25 volatile compounds (Table S1). These compounds were classified into several chemical families, including alcohols, aldehydes, fatty acids, esters, phenolic compounds, furans, and terpenes. The composition and abundance of these compounds reflect the complex metabolic activity of yeast, lactic acid bacteria, and chemical transformations such as Maillard reactions and lipid oxidation occurring during sourdough fermentation. The largest group of compounds were alcohols (7 compounds) and fatty acids (5 compounds). Smaller groups included terpenes (4 compounds), aldehydes (3 compounds), and esters (3 compounds), as well as other minor constituents such as phenolic compounds and furans (a total of 3 compounds).

Among the dominant compounds were alcohols, particularly 1-hexanol, 1-heptanol, and 1-octen-3-ol, which are commonly associated with green, grassy, and mushroom-like aromas. These alcohols may result from lipid oxidation and carbohydrate metabolism, and their presence suggests a fresh and slightly vegetal aroma typical of sourdough systems. Phenylethyl alcohol, an aromatic alcohol with a characteristic floral, rose-like note, was also detected, likely produced by yeast via the Ehrlich pathway.

Aldehydes, such as nonanal, decanal, and dodecanal, contributed to fatty, citrus, and waxy aromas. These long-chain aldehydes are typically formed through oxidation of unsaturated fatty acids and may enhance the sensory complexity of quinoa-based sourdoughs. Furan, 2-pentyl-, a product of lipid oxidation and degradation of linoleic acid, was identified as well and is known to impart beany and green notes.

Fatty acids, including octanoic, nonanoic, decanoic, and tetradecanoic acid, were also present. While essential for microbial metabolism, their soapy, cheesy, or rancid character in higher concentrations may negatively affect sensory acceptance. However, their corresponding esters—such as butyl butanoate, ethyl hexanoate, and ethyl decanoate—were also detected and are known for their pleasant fruity and floral notes, which can mask undesirable odors and improve overall aroma balance.

Notably, terpenes such as D-limonene and eucalyptol, identified mainly in quinoa malt sourdoughs, provided citrus, minty, and herbal nuances. These compounds, commonly associated with plant secondary metabolism, might have originated from the quinoa raw material or been enhanced by germination and fermentation processes.

**Figure 1:**
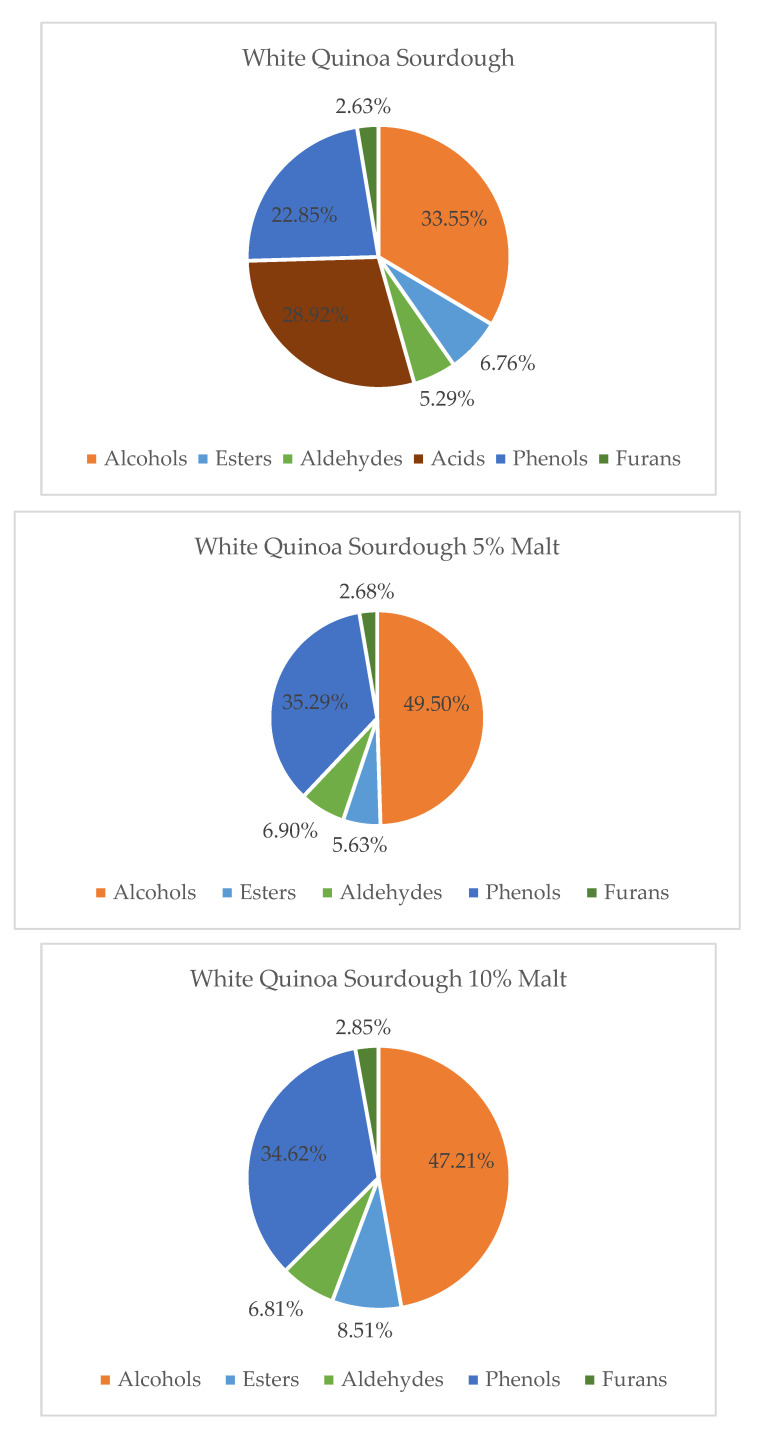
Contribution of various chemical groups in the volatilome of White Quinoa Sourdough and with 5 and 10% of Malts.

**Figure 2:**
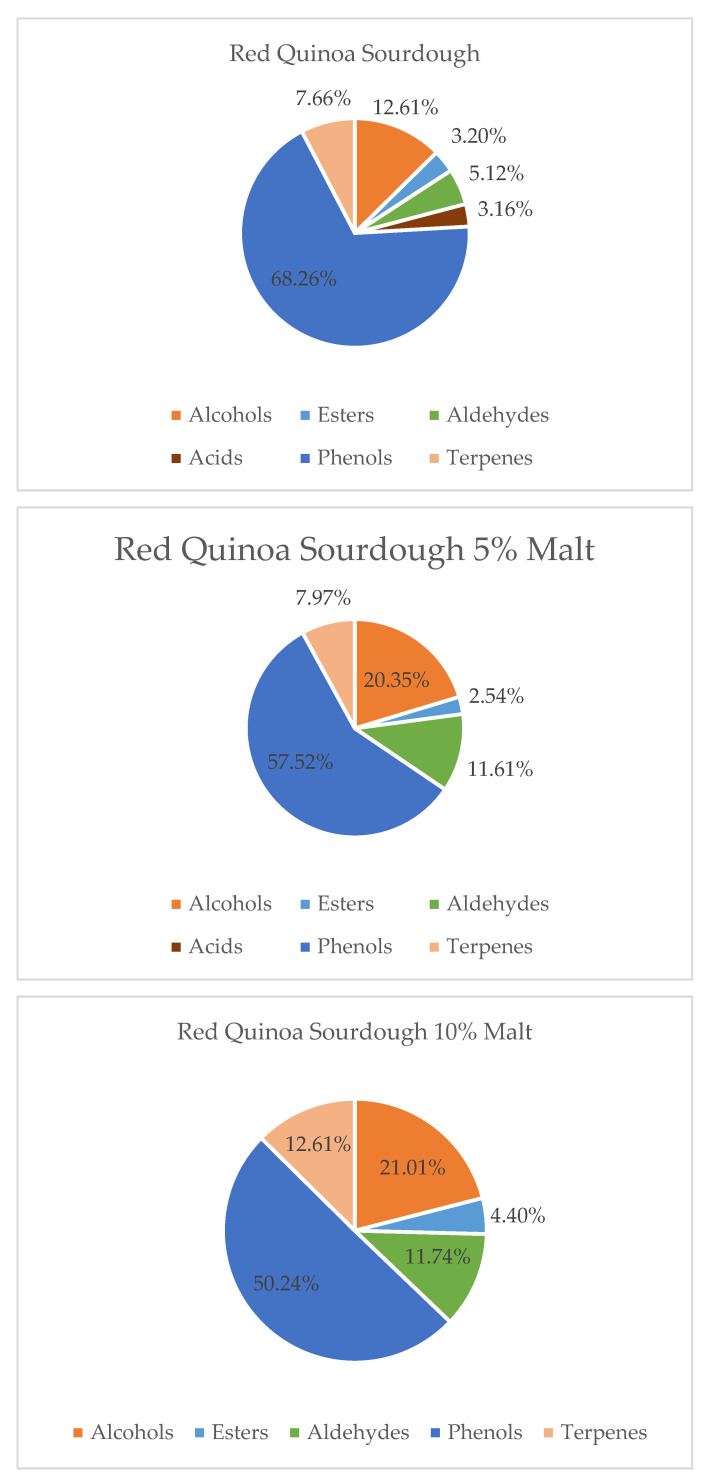
Contribution of various chemical groups in the volatilome of Red Quinoa Sourdough and with 5 and 10% of Malts.

**Figure 3:**
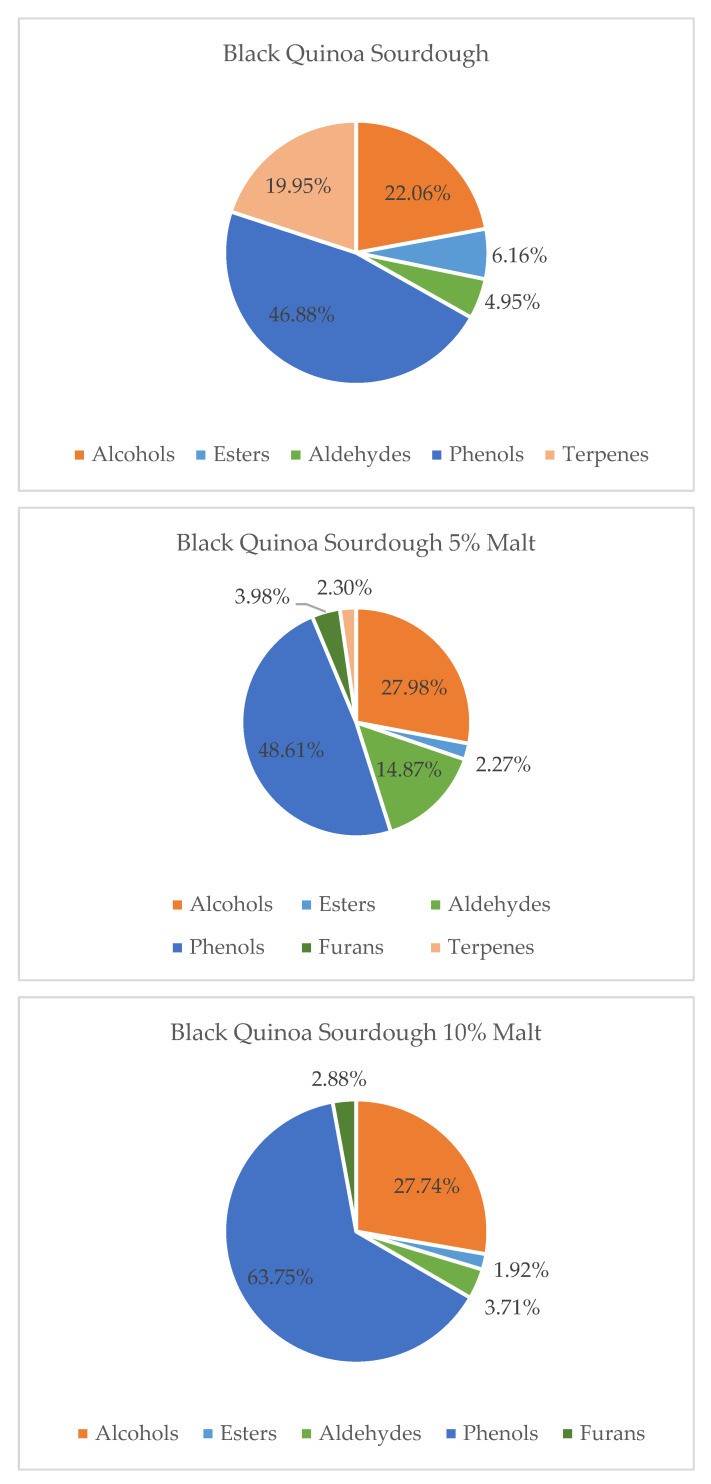
Contribution of various chemical groups in the volatilome of Black Quinoa Sourdough and with 5 and 10% of Malts.

Overall, the volatile composition differed between flour-based and malt-enriched sourdoughs. Samples containing quinoa malts showed a greater abundance of terpenes and esters, contributing to a richer and more complex aroma profile. In contrast, sourdoughs based solely on quinoa flour were dominated by alcohols and fatty acids, yielding more neutral or vegetal notes. These findings align with prior studies on pseudocereal fermentation [8,12,36,37] and emphasize the potential of quinoa malt to enhance sensory quality in gluten-free and functional bakery products. Furthermore, both quinoa variety and malt supplementation level significantly influenced the relative abundance of the main volatile groups—alcohols, acids, esters, aldehydes, phenols, furans, and terpenes—as illustrated in Figure 1, Figure 2 and Figure 3.

Gas chromatography–mass spectrometry (GC–MS) analysis demonstrated that the addition of quinoa malt significantly altered the volatile compound profile of quinoa sourdoughs. The extent and nature of these changes were dependent on both the quinoa variety (white, red, or black) and the percentage of malt addition (5% or 10%). In white quinoa sourdoughs (WQS), malt supplementation led to an increase in the levels of alcohols, aldehydes, esters, and phenolic compounds, which are associated with pleasant sensory attributes, including fruity, floral, and green notes. Concurrently, volatile acids—which can contribute to sharp or undesirable aromas—were relatively reduced. These findings are consistent with prior studies showing that enzymatic activity and microbial metabolism during fermentation enhance the production of volatile compounds with favorable aroma characteristics [38,39,40]. Thus, quinoa malt-enriched WQS may offer enhanced sensory properties for use in gluten-free bakery products [36].

Under the influence of quinoa malt addition to red quinoa sourdough, a decrease in the levels of phenolic compounds and fatty acids was observed, along with an increase in alcohols, aldehydes, esters, and terpenes. These changes led to a significant improvement in the aromatic profile of the sourdough. The rise in alcohols, aldehydes, esters, and terpenes contributed to the development of more intense, pleasant, and complex aromas, such as fruity, citrusy, herbal, and fresh notes. At the same time, the reduction in phenolic compounds and fatty acids helped minimize harsh, bitter, or rancid undertones, resulting in a cleaner and more balanced aroma. These changes suggest that malt processing may modulate precursor availability or metabolic activity during fermentation, leading to the suppression of bitter or oxidized aroma notes [1,12,24,25]. Overall, the addition of quinoa malt enhanced the sensory quality and aromatic appeal of red quinoa sourdough, highlighting its potential for use in functional and flavor-rich bakery products.

In contrast, black quinoa sourdoughs (BQS) showed a more complex response. The addition of quinoa malt to black quinoa sourdough resulted in an increase in alcohols and phenolic compounds, the appearance of furans, and a decrease in terpenes and esters, particularly at the 10% malt level. This suggests a shift toward heavier, more earthy or roasted aromas. A 5% malt addition appeared to offer a better balance by maintaining aldehyde-derived citrus notes while avoiding excessive accumulation of less desirable volatiles. These results highlight the importance of dose optimization when enriching BQS to preserve freshness and aromatic complexity.

Overall, the results confirm that quinoa malt addition modulates the volatilome of sourdoughs in a variety-dependent manner, as confirmed by ANOVA and Duncan’s post hoc test (*p* < 0.05). The enhanced production of esters, aldehydes, and terpenes in malt-enriched WQS and RQS supports their potential application in the formulation of flavorful, functional, and gluten-free baked goods [41]. Conversely, care should be taken in the formulation of BQS products, where excessive malt levels may mask favorable notes with earthy or lipid-derived aromas. The volatile compound profile of quinoa-based sourdoughs markedly differs from that of traditional wheat and rye sourdoughs. Quinoa sourdoughs are characterized by higher levels of alcohols, esters, and terpenes, which contribute to fresh, floral, and herbal aroma notes, whereas wheat and rye sourdoughs are predominantly associated with lactic, malty, and roasted aromatic profiles [42]. These findings contribute to a growing body of literature on pseudocereal-based fermentations and their role in developing novel bakery ingredients with enhanced sensory profiles.

The fermentation profile of quinoa-based sourdoughs, enriched with 5% or 10% quinoa malt, revealed notable differences in carbohydrate degradation and metabolite production depending on both quinoa variety and malt addition. These differences provide insight into the fermentability and microbial activity supported by each matrix. Table 5 summarizes the concentrations of dextrins, glucose, lactic acid, acetic acid, and ethanol across various sample groups, including WQS, RQS, and BQS, with their respective modifications (5%M and 10%M). Dextrin concentrations varied significantly among the samples, ranging from 6.21 ± 0.00 g/kg (RQS5%M) to 8.66 ± 0.003 and 8.67 ± 0.00 g/kg (WQS5%M and BQS10%M). The increased dextrin content in modified samples such as BQS10%M suggests that malting contributed to enhanced starch breakdown into intermediate oligosaccharides, likely through the activation of endogenous amylases during germination. Notably, RQS5%M exhibited a decrease in dextrins, possibly due to rapid microbial utilization or enzymatic hydrolysis progressing further into monosaccharides, a result that aligns with its elevated glucose content.

Glucose concentrations demonstrated the highest variability, with a more than twofold difference between the lowest (WQS5%M: 20.96 ± 0.15 g/kg) and highest (RQS10%M: 47.95 ± 0.38 g/kg) values. Unmodified red quinoa (RQS) already showed a high glucose level (46.71 ± 0.01 g/kg), and its 10% malt-enriched counterpart further increased it slightly. This may be attributed to the naturally higher sugar content in red quinoa and efficient starch saccharification during malting and fermentation. In contrast, BQS and WQS samples showed lower initial glucose, but malting partially increased their concentrations, suggesting a balance between saccharification and microbial consumption.

Lactic acid concentrations were significantly elevated in all modified samples, with BQS10%M reaching the highest value (12.92 ± 0.00 g/kg). These findings indicate that malt supplementation stimulates lactic acid bacteria (LAB) activity, likely by improving substrate availability (e.g., soluble sugars) and fermentation conditions (e.g., acidity, water activity). The increase in LAB metabolites across all varieties after modification supports the role of quinoa malt as a fermentation enhancer, particularly effective in dark quinoa variants.

Acetic acid content, however, followed an inverse trend: it was highest in unmodified WQS (4.07 ± 0.00 g/kg) and decreased with malt addition, particularly in RQS10%M (1.85 ± 0.01 g/kg). This pattern may reflect a shift in microbial metabolism from heterofermentative to homofermentative pathways, potentially favored by increased sugar availability or reduced oxygen levels. The lower acetic acid-to-lactic acid ratio in malted samples may enhance the sensory profile of the final product by reducing vinegar-like notes and promoting mild acidity.

Ethanol concentrations were variable and appeared only in selected samples, primarily within the WQS group. WQS10%M (5.74 ± 0.04 g/kg) and WQS5%M (5.63 ± 0.02 g/kg) had the highest ethanol levels, while ethanol was below detection limits in all modified RQS and BQS samples. This indicates that white quinoa sourdoughs provide a more favorable environment for yeast fermentation, possibly due to lower phenolic content or fewer inhibitory matrix effects compared to red and black quinoa. Alternatively, microbial competition or metabolic rerouting in colored quinoa matrices may reduce ethanol formation. An intriguing observation in this study was the high residual glucose content in red quinoa sourdough accompanied by the low ethanol concentration among the samples. This apparent contradiction suggests a diversion of microbial metabolism away from alcoholic fermentation pathways. One likely explanation is that lactic acid bacteria (LAB), particularly *Lactobacillus* species, preferentially utilized the available glucose for homo- or heterofermentative lactic acid production, rather than facilitating ethanol synthesis via yeast-mediated glycolysis. Red quinoa sourdoughs also exhibited the highest viscosity and SDF, which may have influenced microbial ecology and fermentation kinetics by creating a more favorable environment for LAB dominance over yeasts. Additionally, higher levels of phenolic compounds in red quinoa could exert selective pressure on yeast populations, further tipping the balance toward LAB-driven pathways [41]. These findings imply that in red quinoa systems, glucose is preferentially funneled into organic acid pathways, contributing to acidity and functional value, rather than ethanol accumulation. This shift may be advantageous for sourdoughs aimed at health-conscious or alcohol-sensitive consumers, and warrants further investigation into strain-specific microbial dynamics in colored quinoa fermentations.

Collectively, these ANOVA results show that quinoa variety and malt addition strongly influence the fermentability and metabolic output of sourdough systems. White quinoa with malt supplementation supports higher ethanol and lactic acid production, while black quinoa modifications favor dextrin accumulation and lactic acid yield. Red quinoa exhibits high sugar content but limited ethanol production, suggesting distinctive metabolic responses depending on matrix composition. These insights are essential for designing quinoa-based fermented products with targeted sensory and nutritional properties.

The findings of this study have significant implications for the development of gluten-free and functional bakery products. The enhanced nutritional profiles observed in quinoa malt-enriched sourdoughs—particularly the increased dietary fiber content (up to 18.42 ± 0.10% TDF in BQS10%M), superior mineral density (potassium up to 7896 ± 176 mg/kg, manganese up to 57.62 ± 0.71 mg/kg), and improved protein quality—position these sourdoughs as valuable ingredients for addressing nutritional deficiencies common in gluten-free diets. The complex volatile profiles generated through malt supplementation, characterized by pleasant fruity, floral, and herbal notes from enhanced ester and terpene formation, could help overcome the sensory limitations that often challenge consumer acceptance of gluten-free breads. Furthermore, the increased viscosity (up to 733 ± 5.59 mPa·s) and enhanced fermentation characteristics (TTA up to 23.8 ± 0.2) observed in malted variants suggest improved dough handling properties and bread structure formation, critical factors for commercial bakery applications. The variety-specific responses identified in this study enable targeted formulation strategies: white quinoa sourdoughs for enhanced mineral bioavailability and sensory appeal, red quinoa variants for maximum texture improvement, and black quinoa formulations for premium functional products targeting health-conscious consumers seeking high-fiber, antioxidant-rich alternatives. These quinoa malt sourdoughs thus represent a promising pathway for developing nutritionally superior, commercially viable gluten-free bakery products that meet both dietary restrictions and consumer expectations for taste and texture.

## 3. Materials and Methods

### 3.1. Materials

#### 3.1.1. Raw Material

The study was conducted with three varieties of quinoa (*Chenopodium quinoa* Willd.): white—WQ (Bio Planet S.A., Leszno, Poland), red—RQ (Batom Bio, Kraków, Poland) and black—BQ (Bio Planet S.A., Leszno, Poland). One portion of quinoa grains was grinded to obtain wholemeal quinoa flour, using a KT-120 laboratory hammer type mill (Perten Instruments, Hägersten, Sweden). The second portion underwent a controlled germination and drying process to obtain quinoa malts.

#### 3.1.2. Malting Procedure

Eighty-gram portions of white, red, and black quinoa seeds were weighed and transferred into perforated stainless-steel malting containers, which had been disinfected by drying in a UF110 Plus dryer (Memmert GmbH + Co., Schwabach, Germany) for 2 h at 200 °C, followed by cooling to room temperature. Steeping was performed using a water–air cycle. At the beginning of the process, the containers with seeds were immersed in a 1.5% sodium hypochlorite solution for 10 min to surface-sterilize the seeds, then rinsed three times with distilled water. Subsequently, the containers were submerged in previously boiled and cooled tap water at 18 °C for 4 h, and then transferred to a KK 240 Smart Pro germination chamber (75% relative humidity, 18 °C) for 48 h. The germination time was chosen according to standard practices used in cereal malt production [15]. After germination, each batch was dried in the UF110 Plus dryer at 50 °C for 23 h. White quinoa malt (WQM), red quinoa malt (RQM) and black quinoa malt (BQM) were ground using a KT-120 laboratory hammer-type mill (Perten Instruments, Hägersten, Sweden).

#### 3.1.3. Sourdough Preparation

Sourdoughs were prepared from quinoa flours—white (WQS), red (RQS), and black (BQS)—as control samples, and from blends of each variety with quinoa malts of the same origin added at levels of 5% and 10% (WQS5%M, WQS10%M, RQS5%M, RQS10%M, BQS5%M, BQS10%M). All sourdoughs, with a dough yield of 250, were subjected to spontaneous fermentation at 30 °C for 46 h. The selection of 5% and 10% quinoa malt addition levels was based on preliminary sensory trials and prior literature [17,43]. Higher malt concentrations (>10%) were observed to negatively impact dough handling properties and resulted in excessive acidification, while lower levels (<5%) did not produce detectable differences in aroma or nutritional enhancement. Similarly, the fermentation condition (30 °C for 46 h) was selected to simulate extended fermentation scenarios relevant to traditional sourdough processes and to allow sufficient time for microbial activity to develop. This temperature is within the optimal growth range for lactic acid bacteria and yeasts commonly found in sourdough ecosystems.

### 3.2. Methods

#### 3.2.1. Chemical Composition and Technological Parameters of Quinoa Flours and Malts

Quinoa flours and malts were determined for: falling number according to the Hagberg-Perten method (AACC Method 56-81B) [44], total protein content—with the Kjeldahl method (ICC No. 105/2) [45] using a Foss Tecator Kjeltec 2400 analyzer (Foss, Hilleroed, Denmark) (N × 6.25), moisture ICC No. 110/1 [45], ash content—with the ICC No. 104/1, fat—with the ICC No. 136, soluble (SDF), insoluble (IDF) and total dietary fiber (TDF) (Megazyme kit, Bray, Ireland) acc. AOAC 991.43 method [46], color according to the CIE Lab* color space system.

The samples were analyzed at least in duplicate, and the results are expressed on a dry matter (d.m.) basis.

#### 3.2.2. Determination of Elements in the Quinoa Flours, Malts and Sourdoughs

Sodium (Na), potassium (K), and calcium (Ca) levels were determined using flame emission atomic spectrometry (FEAS), while magnesium (Mg), iron (Fe), copper (Cu), zinc (Zn) and manganese (Mn) concentrations were measured by flame atomic absorption spectrometry (FAAS) using a SpectrAA atomic absorption spectrometer with a Varian AA240FS flame attachment (Varian Inc., Palo Alto, CA, USA) [47,48]. Prior to analysis, all samples were subjected to dry ashing at 550 °C for 8 h. The analyses were performed in a food testing laboratory at the Department of Food Nutrition, University of Environmental and Life Sciences in Wroclaw.

#### 3.2.3. Determination of Physicochemical Properties and Nutritional Composition of Quinoa Sourdough

The pH was measured potentiometrically in a water extract (10 g sample + 90 mL distilled water) using a calibrated digital pH meter (CP-411, Elmetron, Zabrze, Poland), while total titratable acidity (TTA) was determined by titration with 0.1 mol/L NaOH to pH 8.5 and expressed as mL NaOH/10 g sample [2]. The color parameters (L*, a*, b*) were measured using a reflectance colorimeter (CR-400, Konica Minolta, Tokyo, Japan) in the CIE Lab color space after calibration with a white standard [3]. Dynamic viscosity was analyzed using a rotational viscometer (ViscoQC 100, Anton Paar, Graz, Austria) at 25 °C under constant shear conditions, and results were expressed in mPa·s [4]. The total protein content was determined by the Kjeldahl method according to ICC Standard No. 105/2 [5] using a Kjeltec 2400 analyzer (Foss, Hillerød, Denmark), with a nitrogen-to-protein conversion factor of 6.25. Ash content was determined by dry ashing at 550 °C in accordance with ICC No. 104/1 [5]. The contents of total (TDF), soluble (SDF), and insoluble dietary fiber (IDF) were determined enzymatically using the AOAC Method 991.43 and the Megazyme Total Dietary Fiber Assay Kit (Megazyme, Bray, Ireland) [6].

#### 3.2.4. Adsorption of Volatile Compounds Using Solid-Phase Microextraction (SPME)

To enable chromatographic analysis of volatile compounds present in sourdough samples, the volatiles were adsorbed onto a solid-phase microextraction (SPME) fiber [49]. A 2.0 g portion of sourdough was placed into a 20 mL headspace vial. Then, 50 ng of the internal standard (2-undecanone, added as 20 μL of a hexane solution) was introduced. The vial was sealed with a magnetic screw cap fitted with a PTFE/silicone septum.

The septum was pierced with the needle of an SPME holder equipped with a DVB/CAR/PDMS fiber (50/30 μm; Supelco, Bellefonte, PA, USA). The vial was placed on a stand at room temperature (25 °C). After a 5 min equilibration period, the fiber was exposed for 20 min to allow adsorption of volatile compounds onto the fiber coating. Following extraction, the fiber was retracted into the holder prior to GC-MS analysis.

#### 3.2.5. Gas Chromatography–Mass Spectrometry (GC-MS) Analysis

The analysis of volatile compounds was conducted using a GC-2010 Plus gas chromatograph coupled with a GCMS-QP2010 SE mass spectrometer (Shimadzu, Kyoto, Japan). Separation was performed on a ZB-5 capillary column (30 m × 0.25 mm i.d., 0.25 μm film thickness; Phenomenex, Torrance, CA, USA). The injection port temperature was set to 195 °C. Helium was used as the carrier gas at a flow rate of 1.78 mL/min, with an initial inlet pressure of 100 kPa. The oven temperature program was as follows: initial temperature 40 °C (held for 1 min), ramped at 8 °C/min to 195 °C, and held at the final temperature for 5 min. The ion source temperature was maintained at 250 °C, and the interface temperature at 195 °C. Mass spectra were acquired in the scan range of *m*/*z* 35–350 using electron ionization (EI) at 70 eV. The event time was 0.3 s, with a scan speed of 1111. Each sample was analyzed in triplicate.

Volatile compounds separated from quinoa sourdough were identified by mass spectral analysis using the NIST17 chemical standard libraries and, whenever possible, by comparison with spectra and retention times of authentic chemical standards. If an authentic standard was not available, identification was based on a similarity score of at least 95% in the NIST libraries. In cases where authentic standards were employed, the retention time of the analyzed compound could not deviate by more than 0.05 min from that of the standard measured under identical conditions, using the same column and temperature program. Chromatographic peaks were integrated with the use of Shimadzu PostRun Analysis program (Shimadzu, Kyoto, Japan).

#### 3.2.6. Carbohydrate Profile and Fermentation By-Products Analysis

The analysis of the carbohydrate profile—including dextrins (DP4+) and glucose—as well as fermentation by-products such as lactic acid, acetic acid, and ethanol, was performed using high-performance liquid chromatography (HPLC). Approximately 12 g of sourdough sample was weighed and transferred into a flask, followed by the addition of 50 mL of distilled water. The mixture was shaken for 15 min to extract soluble components. The contents were then transferred into a 100 mL volumetric flask, and Carrez reagents were added for protein precipitation. The volume was adjusted to 100 mL with distilled water. The solution was subsequently centrifuged for 20 min, and the supernatant was filtered through a 0.22 µm syringe filter into chromatographic vials.

The analysis was carried out using a Prominence HPLC system (Shimadzu, Kyoto, Japan) equipped with a refractive index detector (RID) and a Rezex ROA-Organic Acid H^+^ column. A 20 µL aliquot of each sample was injected into the column. The mobile phase consisted of 0.005 M sulfuric acid (H_2_SO_4_), with a flow rate of 0.6 mL/min. The column temperature was maintained at 60 °C, and the detector temperature was set at 50 °C.

#### 3.2.7. Statistical Analysis

The results presented are mean values ± standard deviation (SD). Statistical analysis such as one-way ANOVA were analyzed using Statistica 13.3 (StatSoft, Kraków, Poland). Significant differences (*p* ≤ 0.05) between the mean values were determined using Duncan’s Multiple Range Test.

## 4. Conclusions

This study demonstrated that quinoa sourdoughs enriched with quinoa malts exhibit distinct improvements in both nutritional and sensory qualities. The chemical composition analysis revealed that the use of quinoa malt increased dietary fiber content, particularly in black quinoa samples, while also influencing mineral profiles—most notably enhancing manganese, copper, and calcium levels in selected variants. The incorporation of quinoa malt, especially at the 5% level, contributed to increased viscosity, acidity (TTA), and color intensity, which are desirable attributes for sourdough-based products.

Importantly, GC-MS analysis of volatile compounds confirmed that quinoa malt addition modulates the aromatic profile of sourdoughs by enriching the presence of esters, aldehydes, terpenes, and alcohols. These compounds are associated with fruity, floral, and herbal aromas, contributing to a more complex and appealing flavor profile. White and red quinoa sourdoughs enriched with malt showed the most favorable balance of volatiles, while black quinoa sourdough required careful adjustment of malt levels to avoid overpowering, earthy notes.

The combination of quinoa variety and malt percentage had a significant impact on the overall sourdough profile, highlighting the potential to customize fermentation systems to meet specific nutritional and sensory goals. The use of quinoa malt in sourdough fermentation presents a promising strategy to develop functional, gluten-free bakery products with enhanced health benefits and consumer appeal.

## Figures and Tables

**Table 1:** Quality characteristics of white, red, black quinoa flours and their malts.

Sample	Falling Number [s]	Total Protein [%]	Moisture [%]	Ash [%]	Fat [%]	SDF [%]	IDF [%]	TDF [%]	L*	a*	b*
**WQ**	1712 ± 86 ^b^	13.9 ± 0.1 ^e^	11.0 ± 0.0 ^a^	2.41 ± 0.04 ^d^	5.80 ± 0.14 ^e^	2.55 ± 0.01 ^a^	6.65 ± 0.06 ^c^	9.20 ± 0.21 ^d^	89.49 ± 0.22 ^a^	−4.92 ± 0.05 ^e^	16.63 ± 0.24 ^c^
**RQ**	467 ± 23 ^cd^	14.9 ± 0.1 ^c^	10.4 ± 0.1 ^c^	2.32 ± 0.02 ^e^	6.05 ± 0.07 ^d^	3.34 ± 0.03 ^a^	9.24 ± 0.12 ^bc^	12.58 ± 0.51 ^bc^	68.53 ± 0.30 ^e^	0.24 ± 0.09 ^a^	17.85 ± 0.08 ^b^
**BQ**	2091 ± 105 ^a^	16.3 ± 0.1 ^a^	10.9 ± 0.0 ^b^	2.70 ± 0.01 ^a^	7.05 ± 0.07 ^b^	2.86 ± 0.03 ^a^	13.16 ± 0.31 ^a^	16.02 ± 0.63 ^a^	70.79 ± 0.19 ^d^	−2.88 ± 0.04 ^d^	11.41 ± 0.07 ^f^
**WQM**	584 ± 29 ^c^	14.4 ± 0.0 ^d^	3.9 ± 0.1 ^e^	2.51 ± 0.00 ^c^	6.15 ± 0.07 ^d^	1.74 ± 0.00 ^a^	7.28 ± 0.06 ^c^	9.01 ± 0.18 ^d^	88.68 ± 0.01 ^b^	−5.22 ± 0.01 ^f^	19.67 ± 0.28 ^a^
**RQM**	116 ± 6 ^d^	15.2 ± 0.0 ^c^	5.5 ± 0.1 ^d^	2.17 ± 0.03 ^f^	6.50 ± 0.14 ^c^	2.04 ± 0.01 ^a^	8.93 ± 0.09 ^bc^	10.97 ± 0.12 ^cd^	73.56 ± 0.04 ^c^	−0.78 ± 0.01 ^b^	14.74 ± 0.07 ^d^
**BQM**	322 ± 16 ^cd^	16.4 ± 0.2 ^a^	3.1 ± 0.0 ^f^	2.60 ± 0.00 ^b^	7.45 ± 0.07 ^a^	2.90 ± 0.02 ^a^	11.96 ± 0.22 ^ab^	14.86 ± 0.84 ^ab^	71.12 ± 0.03 ^d^	−2.67 ± 0.07 ^c^	12.34 ± 0.09 ^e^

Values are expressed as the mean ± standard deviation. Mean values bearing different letters in the same column denote statistical differences (a > b > c …, etc.) (*p* < 0.05) according to one-way ANOVA followed by Duncan’s post hoc test. WQ—white quinoa, RQ—red quinoa, BQ—black quinoa, WQM—white quinoa malt, RQM—red quinoa malt, BQM—black quinoa malt, SDF—soluble dietary fiber, IDF—insoluble dietary fiber, TDF—total dietary fiber, L*—lightness, ranging from 0 (black) to 100 (white).

**Table 2:** Content of selected minerals in white, red, black quinoa and in their malts.

Sample	Na [mg/kg]	Ca [mg/kg]	K [mg/kg]	Mg [mg/kg]	Cu [mg/kg]	Mn [mg/kg]	Fe [mg/kg]	Zn [mg/kg]
**WQ**	22.08 ± 0.25 ^d^	45.90 ± 3.98 ^b^	5090 ± 94 ^d^	4897 ± 80 ^a^	5.42 ± 0.11 ^b^	24.26 ± 0.60 ^d^	97.54 ± 5.36 ^a^	32.61 ± 0.52 ^c^
**RQ**	8.25 ± 0.37 ^e^	46.32 ± 1.98 ^b^	6384 ± 20 ^b^	4193 ± 28 ^d^	4.50 ± 0.07 ^e^	31.23 ± 0.35 ^c^	103.62 ± 1.64 ^a^	34.79 ± 0.37 ^b^
**BQ**	6.88 ± 0.21 ^e^	50.81 ± 3.20 ^b^	7477 ± 64 ^a^	4333 ± 43 ^c^	4.76 ± 0.02 ^cd^	52.64 ± 0.42 ^b^	83.41 ± 1.78 ^a^	29.22 ± 0.20 ^e^
**WQM**	59.97 ± 0.99 ^a^	57.60 ± 2.99 ^a^	5024 ± 40 ^d^	4961 ± 19 ^a^	6.04 ± 0.38 ^a^	23.63 ± 0.23 ^d^	103.25 ± 8.35 ^a^	34.02 ± 0.86 ^b^
**RQM**	50.53 ± 0.31 ^b^	48.56 ± 2.58 ^b^	5561 ± 77 ^c^	4294 ± 68 ^cd^	4.59 ± 0.06 ^de^	31.80 ± 0.20 ^c^	99.26 ± 2.31 ^a^	36.96 ± 0.69 ^a^
**BQM**	43.16 ± 0.51 ^c^	48.13 ± 5.08 ^b^	6577 ± 76 ^b^	4673 ± 26 ^b^	4.92 ± 0.06 ^c^	53.87 ± 0.88 ^a^	97.90 ± 1.60 ^a^	30.33 ± 0.35 ^d^

Values are expressed as the mean ± standard deviation. Mean values bearing different letters in the same column denote statistical differences (a > b > c …, etc.) (*p* < 0.05) according to one-way ANOVA followed by Duncan’s post hoc test. WQ—white quinoa, RQ—red quinoa, BQ—black quinoa, WQM—white quinoa malt, RQM—red quinoa malt, BQM—black quinoa malt.

**Table 3:** Quality characteristics of white, red, black quinoa sourdoughs with their malts.

Sample	pH	TTA	L*	a*	b*	Dynamic Viscosity [mPa·s]	Total Protein [%]	Ash [%]	SDF [%]	IDF [%]	TDF [%]
**WQS**	4.30 ± 0.00 ^c^	20.2 ± 0.3 ^d^	89.5 ± 0.9 ^a^	−6.09 ± 0.09 ^e^	18.85 ± 0.19 ^c^	595 ± 2.74 ^d^	14.9 ± 0.1 ^e^	2.58 ± 0.02 ^c^	2.28 ± 0.44 ^b^	5.55 ± 0.41 ^d^	7.82 ± 0.32 ^de^
**WQS5%M**	4.33 ± 0.04 ^bc^	23.2 ± 0.3 ^a^	89.0 ± 0.6 ^a^	−5.99 ± 0.07 ^e^	19.46 ± 0.17 ^c^	584 ± 1.78 ^d^	14.5 ± 0.2 ^f^	2.50 ± 0.02 ^d^	2.67 ± 0.19 ^b^	5.82 ± 0.17 ^d^	8.49 ± 0.87 ^d^
**WQS10%M**	4.28 ± 0.01 ^c^	23.8 ± 0.2 ^a^	89.9 ± 0.4 ^a^	−5.75 ± 0.02 ^d^	21.95 ± 0.18 ^a^	666 ± 7.29 ^b^	14.7 ± 0.0 ^ef^	2.60 ± 0.01 ^c^	2.56 ± 0.45 ^b^	4.16 ± 0.14 ^e^	6.73 ± 0.17 ^e^
**RQS**	4.30 ± 0.00 ^c^	20.4 ± 0.3 ^cd^	69.0 ± 0.2 ^c^	0.91 ± 0.02 ^b^	18.90 ± 0.07 ^c^	611 ± 5.78 ^c^	16.0 ± 0.0 ^c^	2.48 ± 0.00 ^d^	4.01 ± 0.07 ^a^	7.60 ± 0.29 ^c^	11.62 ± 0.16 ^c^
**RQS5%M**	4.30 ± 0.01 ^c^	20.0 ± 0.4 ^d^	72.7 ± 0.3 ^b^	1.36 ± 0.08 ^a^	20.25 ± 0.12 ^b^	596 ± 5.72 ^d^	15.8 ± 0.1 ^d^	2.50 ± 0.08 ^d^	3.91 ± 0.21 ^a^	11.88 ± 0.32 ^b^	15.79 ± 0.55 ^b^
**RQS10%M**	4.30 ± 0.02 ^c^	20.4 ± 0.6 ^cd^	69.2 ± 0.2 ^c^	1.05 ± 0.07 ^b^	18.86 ± 0.03 ^c^	733 ± 5.59 ^a^	15.7 ± 0.0 ^d^	2.45 ± 0.02 ^d^	3.38 ± 0.34 ^ab^	11.12 ± 0.17 ^b^	14.50 ± 0.87 ^b^
**BQS**	4.47 ± 0.00 ^a^	20.0 ± 0.7 ^d^	64.1 ± 0.4 ^d^	−2.29 ± 0.03 ^c^	10.15 ± 0.14 ^d^	474 ± 5.59 ^f^	17.3 ± 0.1 ^a^	2.84 ± 0.03 ^a^	2.32 ± 0.14 ^b^	15.62 ± 0.18 ^a^	17.94 ± 0.14 ^a^
**BQS5%M**	4.45 ± 0.07 ^ab^	21.6 ± 0.1 ^b^	61.6 ± 0.5 ^e^	−2.26 ± 0.02 ^c^	9.78 ± 0.05 ^d^	544 ± 6.34 ^e^	17.0 ± 0.1 ^b^	2.78 ± 0.02 ^ab^	2.29 ± 0.01 ^b^	15.42 ± 0.56 ^a^	17.72 ± 0.15 ^a^
**BQS10%M**	4.40 ± 0.14 ^abc^	21.2 ± 0.2 ^bc^	62.4 ± 0.1 ^e^	−2.14 ± 0.04 ^c^	10.20 ± 0.09 ^d^	667 ± 6.20 ^b^	16.9 ± 0.1 ^b^	2.76 ± 0.02 ^b^	3.35 ± 0.18 ^ab^	15.07 ± 0.28 ^a^	18.42 ± 0.10 ^a^

Values are expressed as the mean ± standard deviation. Mean values bearing different letters in the same column denote statistical differences (a > b > c …, etc.) (*p* < 0.05) according to one-way ANOVA followed by Duncan’s post hoc test. WQS—white quinoa sourdough, WQS5%M—white quinoa sourdough with 5% of white quinoa malt, WQS10%M—white quinoa sourdough with 10% of white quinoa malt, RQS—red quinoa sourdough, RQS5%M—red quinoa sourdough with 5% of red quinoa malt, RQS10%M—red quinoa sourdough with 10% of red quinoa malt, BQS—black quinoa sourdough, BQS5%M—black quinoa sourdough with 5% of black quinoa malt, BQS10%M—black quinoa sourdough with 10% of black quinoa malt.

**Table 4:** Content of selected minerals in white, red, black quinoa sourdoughs with their malts.

Sample	Na [mg/kg]	Ca [mg/kg]	K [mg/kg]	Mg [mg/kg]	Cu [mg/kg]	Mn [mg/kg]	Fe [mg/kg]	Zn [mg/kg]
**WQS**	77.06 ± 0.86 ^b^	41.91 ± 0.78 ^b^	6116 ± 104 ^c^	5371 ± 70 ^a^	5.84 ± 0.05 ^b^	26.53 ± 0.19 ^f^	102.37 ± 1.34 ^a^	34.49 ± 0.26 ^cd^
**WQS5%M**	80.95 ± 2.16 ^a^	46.72 ± 0.65 ^a^	5851 ± 65 ^cd^	5204 ± 58 ^ab^	6.16 ± 0.11 ^a^	26.00 ± 0.16 ^fg^	95.64 ± 0.92 ^a^	35.43 ± 0.91 ^c^
**WQS10%M**	74.82 ± 2.73 ^b^	40.58 ± 1.23 ^b^	5583 ± 127 ^d^	4967 ± 189 ^bc^	5.63 ± 0.07 ^c^	25.62 ± 0.86 ^g^	93.47 ± 1.53 ^a^	34.38 ± 0.64 ^d^
**RQS**	64.53 ± 2.03 ^c^	41.76 ± 1.53 ^b^	7039 ± 194 ^b^	4522 ± 79 ^de^	4.99 ± 0.15 ^d^	34.45 ± 0.38 ^d^	90.76 ± 1.14 ^a^	37.83 ± 1.14 ^a^
**RQS5%M**	61.92 ± 2.33 ^cd^	29.37 ± 1.29 ^c^	6890 ± 148 ^b^	4554 ± 114 ^de^	4.84 ± 0.09 ^e^	33.47 ± 0.33 ^e^	95.98 ± 1.71 ^a^	36.70 ± 0.26 ^b^
**RQS10%M**	59.72 ± 0.41 ^de^	25.48 ± 1.53 ^cd^	6850 ± 104 ^b^	4485 ± 145 ^e^	4.87 ± 0.02 ^de^	33.87 ± 0.28 ^de^	95.42 ± 0.99 ^a^	38.08 ± 0.26 ^a^
**BQS**	57.38 ± 1.36 ^e^	26.81 ± 1.54 ^cd^	7896 ± 176 ^a^	4850 ± 70 ^cd^	4.90 ± 0.08 ^de^	55.65 ± 0.47 ^b^	101.95 ± 1.44 ^a^	31.12 ± 0.57 ^e^
**BQS5%M**	53.92 ± 1.10 ^f^	18.89 ± 1.53 ^e^	7663 ± 179 ^a^	4749 ± 109 ^cde^	4.79 ± 0.09 ^e^	53.90 ± 0.75 ^c^	102.55 ± 1.35 ^a^	30.97 ± 0.62 ^e^
**BQS10%M**	56.65 ± 0.47 ^ef^	23.24 ± 1.63 ^d^	7572 ± 109 ^a^	4814 ± 24 ^cde^	4.89 ± 0.05 ^de^	57.62 ± 0.71 ^a^	106.17 ± 1.05 ^a^	31.36 ± 0.78 ^e^

Values are expressed as the mean ± standard deviation. Mean values bearing different letters in the same column denote statistical differences (a > b > c …, etc.) (*p* < 0.05) according to one-way ANOVA followed by Duncan’s post hoc test. WQS—white quinoa sourdough, WQS5%M—white quinoa sourdough with 5% of white quinoa malt, WQS10%M—white quinoa sourdough with 10% of white quinoa malt, RQS—red quinoa sourdough, RQS5%M—red quinoa sourdough with 5% of red quinoa malt, RQS10%M—red quinoa sourdough with 10% of red quinoa malt, BQS—black quinoa sourdough, BQS5%M—black quinoa sourdough with 5% of black quinoa malt, BQS10%M—black quinoa sourdough with 10% of black quinoa malt.

**Table 5:** Concentrations of dextrins, glucose, lactic acid, acetic acid, and ethanol in white, red, black quinoa sourdoughs with their malts.

Sample	Dextrins [g/kg]	Glucose [g/kg]	Lactic Acid [g/kg]	Acetic Acid [g/kg]	Ethanol [g/kg]
WQS	7.86 ± 0.07 ^d^	32.61 ± 0.44 ^d^	7.56 ± 0.02 ^h^	4.07 ± 0.00 ^a^	3.28 ± 0.02 ^c^
WQS5%M	8.66 ± 0.03 ^a^	20.96 ± 0.15 ^i^	12.70 ± 0.02 ^b^	2.56 ± 0.01 ^f^	5.63 ± 0.02 ^b^
WQS10%M	8.48 ± 0.01 ^b^	21.55 ± 0.12 ^h^	12.70 ± 0.01 ^b^	2.42 ± 0.02 ^g^	5.74 ± 0.04 ^a^
RQS	7.43 ± 0.01 ^f^	46.71 ± 0.01 ^b^	10.84 ± 0.00 ^f^	n.d.	3.24 ± 0.01 ^d^
RQS5%M	6.21 ± 0.00 ^g^	39.35 ± 0.00 ^c^	10.68 ± 0.00 ^g^	2.96 ± 0.00 ^c^	n.d.
RQS10%M	8.28 ± 0.00 ^c^	47.95 ± 0.38 ^a^	12.68 ± 0.01 ^b^	1.85 ± 0.01 ^h^	n.d.
BQS	7.68 ± 0.00 ^d^	25.39 ± 0.00 ^g^	11.33 ± 0.01 ^e^	2.79 ± 0.01 ^e^	3.18 ± 0.00 ^e^
BQS5%M	8.56 ± 0.00 ^b^	30.36 ± 0.00 ^f^	12.22 ± 0.01 ^c^	2.96 ± 0.00 ^c^	n.d.
BQS10%M	8.67 ± 0.00 ^a^	31.22 ± 0.00 ^e^	12.92 ± 0.00 ^a^	3.06 ± 0.02 ^b^	n.d.

Values are expressed as the mean ± standard deviation. Mean values bearing different letters in the same column denote statistical differences (a > b > c …, etc.) (*p* < 0.05) according to one-way ANOVA followed by Duncan’s post hoc test. WQS—white quinoa sourdough, WQS5%M—white quinoa sourdough with 5% of white quinoa malt, WQS10%M—white quinoa sourdough with 10% of white quinoa malt, RQS—red quinoa sourdough, RQS5%M—red quinoa sourdough with 5% of red quinoa malt, RQS10%M—red quinoa sourdough with 10% of red quinoa malt, BQS—black quinoa sourdough, BQS5%M — black quinoa sourdough with 5% of black quinoa malt, BQS10%M — black quinoa sourdough with 10% of black quinoa malt, n.d.—not determined.

## Data Availability

Data is contained within the article.

## Supplementary Materials

The following supporting information can be downloaded at: https://www.mdpi.com/article/10.3390/molecules30173653/s1, Table S1. Volatile compounds identified in quinoa sourdoughs with retention times, odor thresholds, odor descriptors, and corresponding references. References [50,51,52,53,54,55,56,57,58,59,60,61,62,63,64,65,66,67,68,69,70,71,72] are cited in the supplementary materials.
